# Design and Study of a Two-Dimensional (2D) All-Optical Spatial Mapping Module

**DOI:** 10.3390/s24072219

**Published:** 2024-03-30

**Authors:** Zhenyu Ma, Haili Yu, Kai Cui, Yang Yu, Chen Tao

**Affiliations:** 1 Suzhou Institute of Biomedical Engineering and Technology, Chinese Academy of Sciences, Suzhou 215163, China; emozhiai123@163.com (Z.M.); cuik@sibet.ac.cn (K.C.); shandian98@163.com (Y.Y.); 2 Changchun Institute of Optics and Fine Mechanics and Physics, Chinese Academy of Sciences, Changchun 130033, China; taochen_jlu@foxmail.com

**Keywords:** all-optical mapping photography, two-dimensional, optical design, periscope array

## Abstract

Sequentially timed all-optical mapping photography is one of the main emerging ultra-fast detection technologies that can be widely applicable to ultra-fast detection at the picosecond level in fields such as materials and life sciences. We propose a new optical structure for an all-optical spatial mapping module that can control the optical field of two-dimensional imaging while improving spectral resolution and detector sensor utilization. The model of optical parameters based on geometrical optics theory for the given structure has been established, and the theoretical analysis of the inter-frame energy crosstalk caused by incident beam spot width, chromatic aberration, and main errors of the periscope array has been conducted. The optical design of the two-dimensional (2D) all-optical spatial mapping module was finally completed using ZEMAX OpticStudio 2018 software. The results show that our optical module can realize targets of 16 frames and 1.25 nm spectral resolution.

## 1. Introduction

All-optical mapping photography [1,2,3] has the advantages of high time resolution, high luminous flux, and anti-radiation interference and can effectively overcome the shortcomings of the pump-probe technique [4,5], which is unable to perform non-repeatable or difficult-to-replicate event observation. Meanwhile, it can also avoid the drawbacks of low spatial resolution and complex image reconstruction from compressed ultra-fast spectroscopy [6,7] and multi-spectral tomography [8]. This technology has significant application prospects in the fields of observation and analysis of events at the picosecond and even femtosecond scale, such as shockwave dynamics, engine tail flame detection, superconducting electronic states, structural dynamics of chemical reactions, protein folding, and the process of photosynthesis [9,10,11,12,13,14].

Amplitude-modulated all-optical mapping photography uses grating and amplitude modulation elements to control the optical field. It is a little more complex in optical structure and has a few frames and low utilization of detection sensor, which is limited by the one-dimensional control mode. However, it has the advantages of high energy utilization, high amplitude consistency, low component fabrication challenge, spatial distribution consistency (image position, spacing, and size), and flexible change of spectral range, which has gradually made it a research hotspot.

Nakagawa et al. first proposed sequentially timed all-optical mapping photography; they introduced the composition of the whole system, which used a one-dimensional periscope array in the spatial mapping module. The principal system was built, and the observation of a 200-fs ultra-fast event was realized, which was published in the high-impact journal *Nature Photonics* [1]. On this basis, they applied this system to observe plasma and phonon polarization dynamics [15]. Subsequently, temporal characteristics, such as exposure time and frame rate, were investigated [16]. Although the principal system has successfully achieved hundred-femtosecond-scale observation, there are problems, such as a small number of frames and low utilization of detectors. Later, diffuse optical elements that may achieve 25-frame burst imaging were proposed for sequentially timed all-optical mapping photography using a spectral filtering technique [17,18]. This system has the advantages of fewer optical elements and a compact structure; however, the fabrication of diffractive optical elements was challenging and expensive, and lower energy utilization and poor consistency of 2D diffraction efficiency limit the further development of this system. Saiki et al. used slicing mirrors instead of the periscope array for complete optical field control [19], which was then developed by Yuan et al. [2,20,21]. This can effectively increase the frame and detector utilization of the instrument; however, there are problems, such as the high precision of the grating and the slice mirror and the existence of chromatic aberration that reduces the quality of imaging.

A higher spectral resolution represents a shorter frame interval, and a higher number of frames means a higher number of observation windows. Furthermore, consistency in frame quality can ensure that all viewing windows are consistent. While amplitude-modulated examples have some inherent advantages, the one-dimensional structure limits the number of increased frames, and wastes another dimension of the ultra-fast image sensor. In summary, we select the periscope array, which is much less challenging to fabricate by combining conditions, as an amplitude modulation element. In order to solve the problems of fewer frames, lower spectral resolution, and lower detector sensor utilization, an optical structure for a two-dimensional (2D) all-optical spatial mapping module is proposed. A parametric model of our optical structure is established verified by Zemax OpticStudio 2018 software. A numerical theoretical calculation is conducted for the inter-frame energy crosstalk, chromatic aberration, and main errors of the periscope array, which cannot be obtained in a non-sequential design.

## 2. Theoretical Design

### 2.1. Composition of All-Optical Spatial Mapping Module

**Figure 1:**
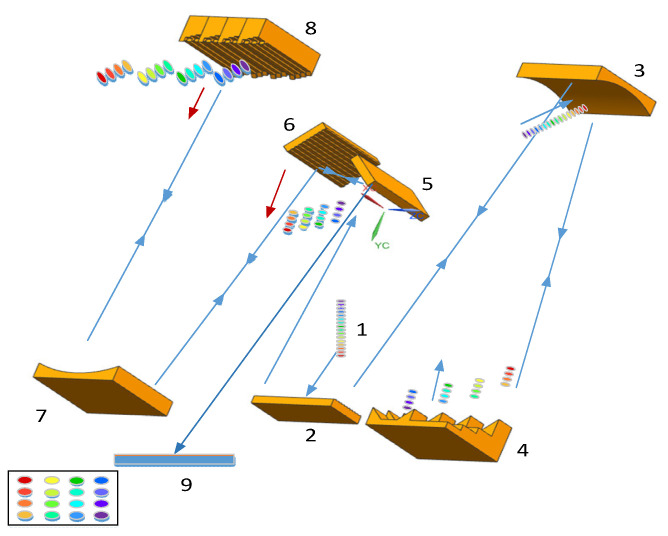
Schematic diagram of two-dimensional (2D) all-optical spatial mapping module.The arrow indicates the direction of light propagation, and the digital label represents the optical element that the light incident on successively: 1-incident pulsed light; 2-grating; 3-No.1 cylindrical mirror; 4-No. 1 periscope array; 5-mirrors; 6-grating; 7-No.2 cylindrical mirror; 8-No. 2 periscope array; 9-Detector.

The schematic diagram of a two-dimensional (2D) all-optical spatial mapping module is shown in Figure 1. The whole device consists of two orthogonal modulation modules, which include a one-dimensional diffraction grating, a cylindrical mirror, and a periscope array.

**Figure 2:**
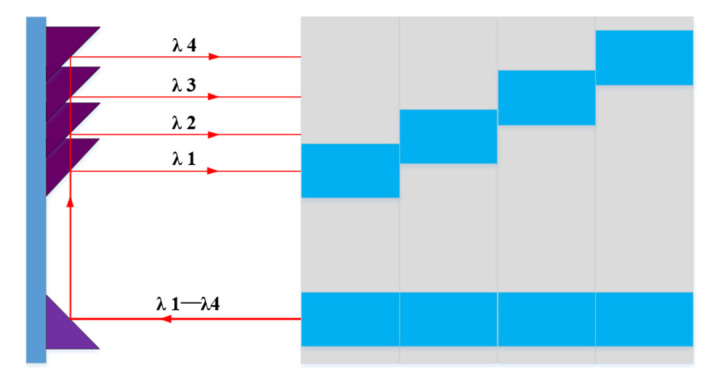
Schematic diagram of the periscope array with top view and front view.

When the incident pulse is diffracted by grating dispersion and reflected by a cylindrical mirror, a series of transverse *λ*_1_–*λ*_4_ spots is formed. The No. 1 periscope array shown in Figure 2 is composed of several array elements fixed together, and each single array is fixed on a flat substrate by a pair of triangular mirrors (with increasing spacing). The difference in thickness of the flat substrate is used to compensate for the optical path variation caused by the difference in distance of the triangular mirrors on different arrays. The *λ*_1_–*λ*_4_ spot is modulated by the No. 1 periscope array and then returned to the diffraction grating via the cylindrical mirror. In the incident direction of the original grating, there is the transverse spatially separated *λ*_1_–*λ*_4_ frame, and then it is incident to another group of the orthogonal spatial mapping module. The No. 2 periscope array is a modular splice of the No. 1 periscope array, which is orthogonal. In the same way, *λ*_1_ is subdivided into *λ*_11_, *λ*_12_, *λ*_13_, *λ*_14_ by grating dispersion and then modulated by the first module of the No. 2 periscope array, and the rest *λ*_2_, *λ*_3_, *λ*_4_ are also modulated in the corresponding module. Finally, a 4 × 4 two-dimensional frame image is formed at the detector by the cylindrical mirror reflection and diffraction grating dispersion combining beam.

### 2.2. Theoretical Design of Two-Dimensional (2D) All-Optical Spatial Mapping Module

**Figure 3:**
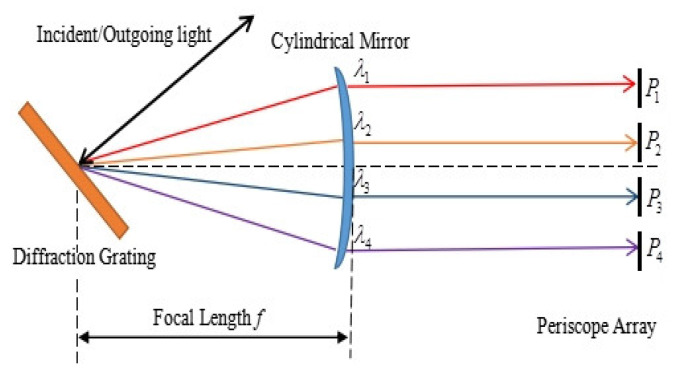
Schematic diagram of one-dimensional spatial mapping module.

The schematic diagram of the one-dimensional spatial mapping module is shown in Figure 3. The cylindrical mirror is equivalent to the transmissive mirror, and separation points P_1_, P_2_, P_3_, and P_4_ on the periscope array correspond to the wavelengths *λ*_1_–*λ*_4_ dispersed by diffraction grating.

According to the grating equation, the diffraction angle *θ*_0_ can be expressed as

(1) $$\theta_{o}=\arcsin\left(\frac{m\lambda }{d}-\sin\theta_{i}\right)$$

In Equation (1), where *d* is the grating constant, *θ_i_* is the angle of incidence, *m* is the diffraction level, *λ* is the wavelength, and *f* is the focal length of the cylindrical mirror, then the spatial distance between two neighboring wavelengths on the periscope array is:

(2) $$\delta_{2}=f\left(\arcsin\left(\frac{m\lambda_{2}}{d}-\sin\theta_{i}\right)-\arcsin\left(\frac{m\lambda_{1}}{d}-\sin\theta_{i}\right)\right)$$

Let *δ = w*. The relationship between the cylindrical mirror and the grating parameters can be expressed as follows:

(3) $$f=\delta \cdot {\left(\arcsin\left(\frac{m\lambda_{2}}{d}-\sin\theta_{i}\right)-\arcsin\left(\frac{m\lambda_{1}}{d}-\sin\theta_{i}\right)\right)}^{-1}$$

**Figure 4:**
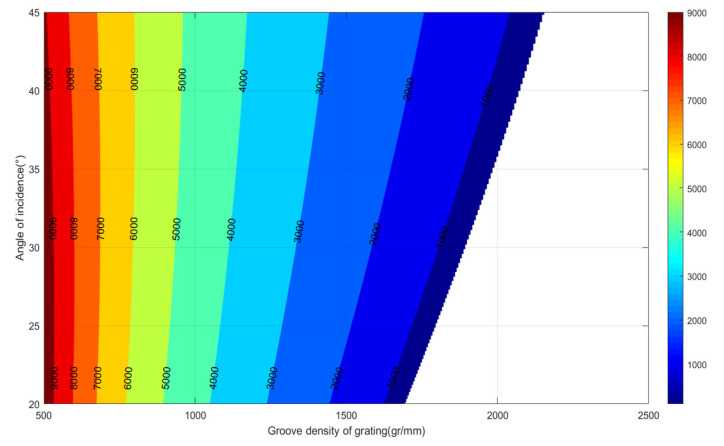
Relation curve of grooving density of grating *1/d*, angle of incidence *θ_i_* and focal length *f* of cylindrical mirror.

According to Equation (3), the grating constant *d*, the angle of incidence *θ_i_*, and the relationship with the focal length of the cylindrical mirror *f* can be determined. Let *w*_0_ = 6 mm, *m* = 1, *λ*_1_ = 790 nm, *λ*_2_ = 791.25 nm, and set the groove density of grating in the range of 500–2500 g/mm, and angle of incidence in the range of 20°–45°. The relation curve is plotted as shown in Figure 4, where the horizontal coordinate is the density of the grating lines, the vertical coordinate is the grating angle of incidence, and the contour line indicates the focal length of the cylindrical mirror. As can be seen from Figure 4, the focal length along with an increased density of grating lines decreases under the same grating angle of incidence, while the focal length along with an increased grating angle of incidence increases under the same density of grating lines.

The colored part in Figure 4 can meet the 6 mm line dispersion requirement and, considering the factors of fabrication difficulty, grating diffraction efficiency and component position interference, the final selected parameters are shown in Table 1:

The value of the width *b* of a single mirror on the periscope array can be expressed as:

(4) $$b_{N}=\frac{\delta_{N}}{2}$$

By using the grating and cylindrical mirror parameters as in Table 1, the b values of the No. 1 and No. 2 periscope array can be calculated as shown in Table 2 according to Equations (2) and (4):

### 2.3. Error Analysis of Two-Dimensional (2D) All-Optical Spatial Mapping Module

#### 2.3.1. Analysis of Inter-Frame Energy Crosstalk Caused by Incident Beam Spot Width

**Figure 5:**
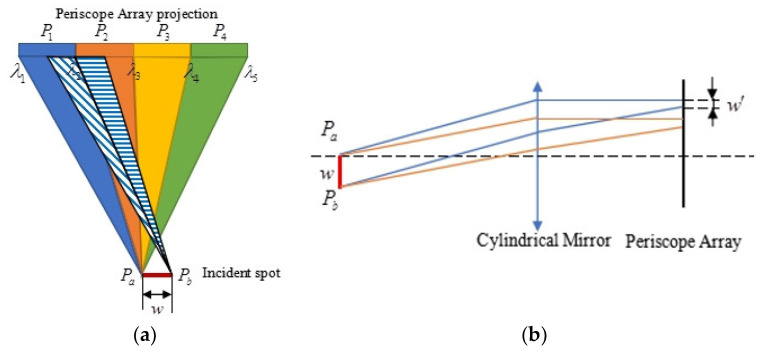
(**a**) Schematic diagram of energy crosstalk at the vertical optical path cross-section; (**b**) schematic diagram of energy crosstalk at the cross-section of the optical path.

When there is a width of incident spot rather than a point, inter-frame energy crosstalk will affect the imaging quality and spectral resolution of the module. The principal schematic is shown in Figure 5.

As shown in Figure 5a, in the plane formed by the direction of the grating line and the grating normal, the incident spot is projected as a line segment with projection size w, and the two endpoints are P_a_ and P_b_. The parameters of the grating and the cylindrical mirrors above are designed based on the point of P_a_, and the P_1_, P_2_, P_3_, and P_4_ points on the periscope array correspond to the wavelengths of *λ*_1_, *λ*_2_, *λ*_3_, and *λ*_4_, which are based on the dispersion of P_a_. The wavelengths *λ*_1_–*λ*_4_ no longer correspond to P_1_–P_4_ points on the periscope array for the other endpoint P_b_.

**Figure 6:**
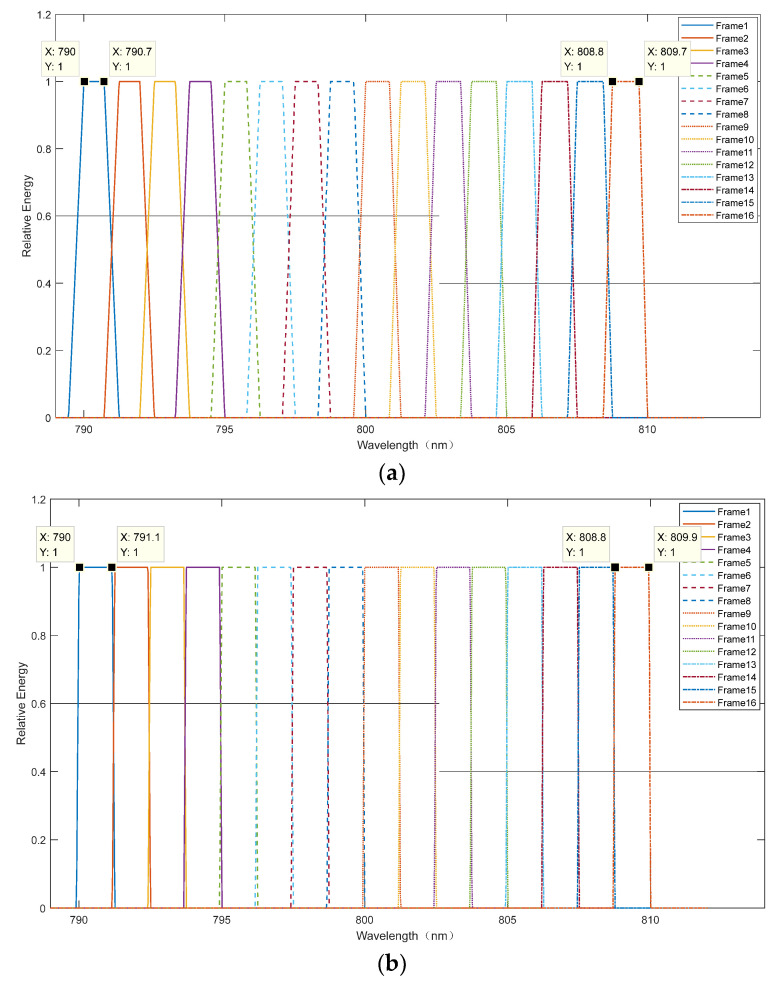
(**a**) Energy distribution of each frame when *w′ = w*; (**b**) energy distribution of each frame when *w′ = w*/6.

The equivalent optical structure is shown in Figure 5b, where *w′* represents offset at any wavelength. We set the offset *w′* equal to *w*, and then the energy distribution of each frame is shown in Figure 6a, where the groove density of the grating is 1800 g/mm, the angle of incidence is 30°, and the focal length of the cylindrical mirror is 1067.5 mm. The analysis above ignores the diffraction efficiency of the grating and the reflectivity of each mirror surface. The horizontal coordinates represent wavelength, the vertical coordinates represent normalized energy, and the curves of different colors represent the normalized energy of each wavelength in a different frame. The flat top indicates the wavelength range without crosstalk; in Figure 6a, its ratio to the design bandwidth (1.25 nm) is between 56% and 72%. We believe the ideal situation is when the ratios are all greater than 85%. While the offset *w′* is equal to *w*/6, the energy distribution of each frame is shown in Figure 6b, and the ratio is between 88% and 96%, which can better eliminate the effect of inter-crosstalk.

It should be additionally pointed out that, when *w′ = 0*, the wavelengths *λ* corresponding to the spots P_a_ to P_b_ are all incident on the same position of the periscope array. At this time, the positions of the incident spot, the cylindrical mirror, and the periscope array satisfy the 4*f* condition, in which inter-frame crosstalk no longer exists.

#### 2.3.2. Analysis of Chromatic Aberration due to Incident Spot Width

**Figure 7:**
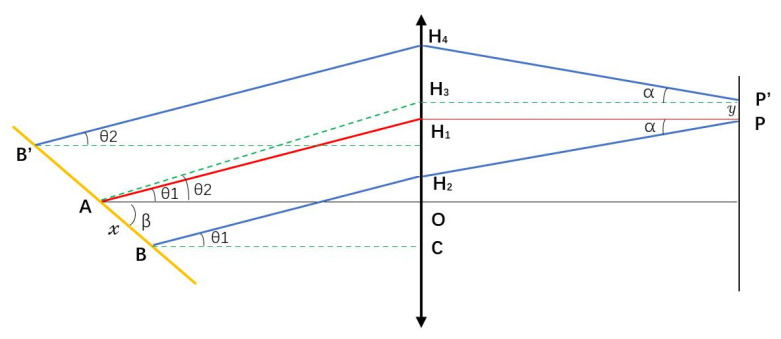
Schematic diagram of imaging characteristics of off-axis points of incident light spots.

Considering the characteristics of the periscope array, off-axis point imaging will cause object—image flip, which results in the spatial position of the image in each frame, so that the image converging at the grating again does not satisfy the achromatic condition. The actual optical path from the grating and the cylindrical mirrors to the periscope array is shown in Figure 7.

The distance of PP’ is the dispersion direction shift on the focal plane, which is set to be *y*, and the distance of *AB* is set to be *x*. Then we have:

(5) $$H_{1}H_{2}=H_{1}O-H_{2}O=H_{1}O-\left(H_{2}C-OC\right)$$

in which:

(6) $$H_{1}O=f\tan\theta_{1}\;H_{2}C=\left(f-x\cos\beta \right)\tan\theta_{1}\;OC=x\sin\beta$$

Coupling gives rise to:

(7) $$H_{1}H_{2}=x\left(\cos\beta \tan\theta_{1}+\sin\beta \right)$$

The relationship between *θ*_1_ and *θ*_2_ is:

(8) $$\tan\theta_{2}=\frac{\left(f\tan\theta_{1}+y\right)}{f}=\tan\theta_{1}\frac{y}{f}$$

in which:

(9) $$y=L_{1}\tan\alpha \;\tan\alpha =\frac{x}{f}\left(\cos\beta \tan\theta_{1}+\sin\beta \right)$$

where *L*_1_ is the offset distance in vertical direction after reflection by the periscope array, and α is the angle between light ray and the normal direction of the periscope array. Then we can obtain:

(10) $$\tan\theta_{2}=\tan\theta_{1}\frac{L_{1}x}{f^{2}}\left(\cos\beta \tan\theta_{1}+\sin\beta \right)$$

For the position of the image as *B′*:

(11) $$\begin{array}{ll} AB^{\prime } & =x\frac{\tan\theta_{1}+\tan\beta }{\tan\theta_{1}+\tan\beta +\frac{y}{f}}\\ & =x\frac{\tan\theta_{1}+\tan\beta }{\tan\theta_{1}+\tan\beta +\frac{L_{1}x}{f^{2}}\left(\cos\beta \tan\theta +\sin\beta \right)} \end{array}$$

The distance between the *B′* point position and point *A* can be determined using Equation (11), when the focal length *f ^2^* » *L*_1_*x*, the outgoing and incident light rays satisfy the grating equation at the same time, and there is no chromatic aberration in our module. We can obtain:

(12) $$\beta =\frac{\pi }{2}-\arcsin\left(\frac{m\lambda_{800nm}}{d}-\sin\theta_{i}\right)$$

where *λ*_800 nm_ is the central wavelength, and for the other wavelengths:

(13) $$\begin{array}{ll} \theta_{1}= & \frac{\pi }{2}-\arcsin\left(\frac{m\lambda }{d}-\sin\theta_{i}\right)-\beta \\ & =\arcsin\left(\frac{m\lambda_{800nm}}{d}-\sin\theta_{i}\right)-\arcsin\left(\frac{m\lambda }{d}-\sin\theta_{i}\right) \end{array}$$

From connective Equations (8) and (13), we can obtain:

(14) $$\begin{array}{ll} \tan\theta_{2} & =\tan\left(\arcsin\left(\frac{m\lambda_{800nm}}{d}-\sin\theta_{i}\right)-\arcsin\left(\frac{m\lambda }{d}-\sin\theta_{i}\right)\right)\\ & +\frac{L_{1}x}{f^{2}}\sin\left(\arcsin\left(\frac{m\lambda_{800nm}}{d}-\sin\theta_{i}\right)\right)\\ & \times \left(\tan\left(\arcsin\left(\frac{m\lambda_{800nm}}{d}-\sin\theta_{i}\right)-\arcsin\left(\frac{m\lambda }{d}-\sin\theta_{i}\right)\right)+\cot\left(\arcsin\left(\frac{m\lambda_{800nm}}{d}-\sin\theta_{i}\right)\right)\right) \end{array}$$

**Figure 8:**
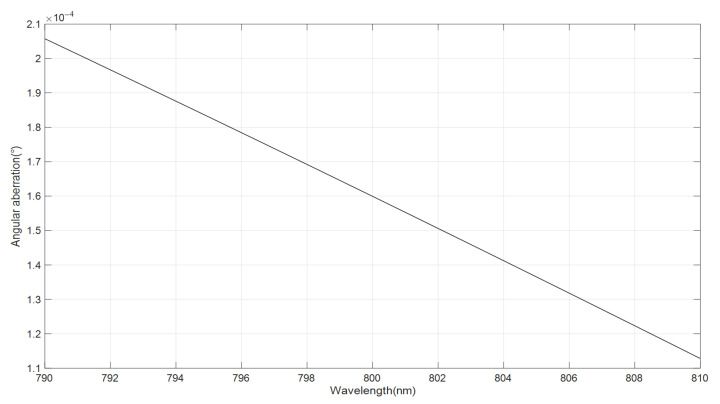
Relation curve of wavelength *λ* and angle aberration after beam combination Δ*θ*.

Let the grooving density of the grating *1/d =* 1800 g/mm, angle of incidence *θ_i_* = 30°*, L*_1_ = 8 mm, and *x* = 3 mm. Different wavelengths are diffracted by the grating and reflected back by the periscope array and cylindrical mirror. Its angle aberration after beam combination (Δ*θ*) curve, between the outgoing angle after beam combination and the angle of incidence, is shown in Figure 8.

As can be seen from Figure 8, the angular aberration Δ*θ* is within 0.0002057° under the condition of 1075 mm focal length, so chromatic aberration can be expressed as:

(15) l=DtanΔθ

where *D* is the total optical path, which is approximately equal to 4*f*, and finally we can compute the maximum chromatic aberration, which is equal to 15.44 um.

#### 2.3.3. Analysis of Main Errors of the Periscope Array

Because of the long focal length of the module, any error of the periscope array may be more stringent, and the following analysis is carried out for the main errors of the periscope array fabrication.

- Substrate angle error of the periscope array

**Figure 9:**
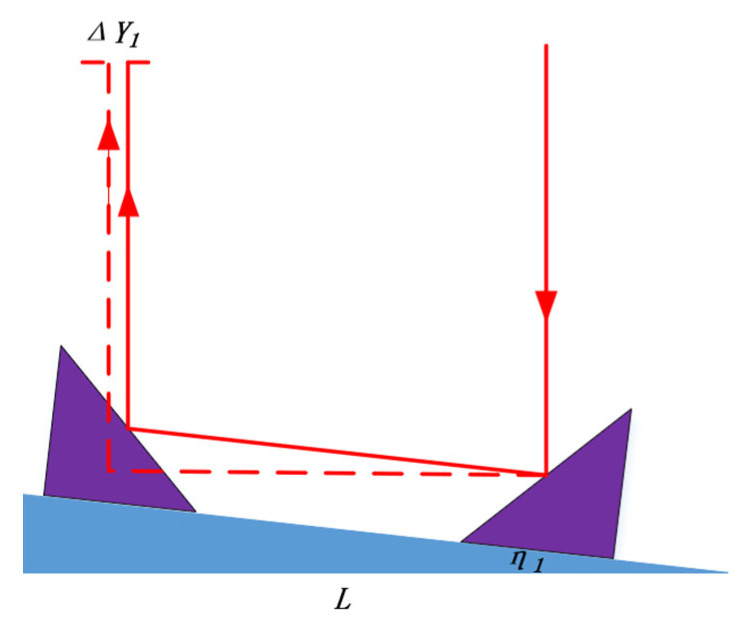
Schematic diagram of substrate angle error of the periscope array. The arrow indicates the direction of light propagation. The dashed line represents the ideal propagation direction, and the solid line represents the actual propagation direction when there is an error.

The spot is shifted when there is a substrate angle error on the periscope array, and Figure 9 shows a schematic diagram of a substrate angle error:

(16) $$\Delta Y_{1}=L\times \sin^{2}\eta_{1}$$

where *L* represents the lateral distance from the midpoint of the step reflection unit to the rightmost side of the step, and *η*_1_ is the angular deviation as in Figure 9.

- Triangular reflector traverse error of the periscope array

**Figure 10:**
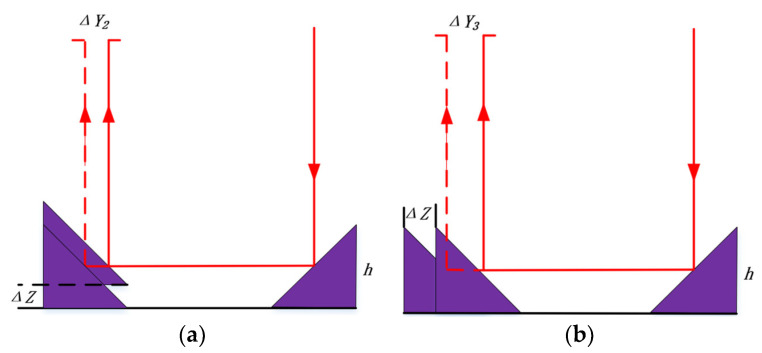
(**a**) Schematic diagram of the triangular reflector traverse error of the periscope array in the direction of the outgoing light; (**b**) Schematic diagram of the triangular reflector traverse error of the periscope array in the vertical direction of the outgoing light.

As shown in Figure 10a,b, the periscope array has a triangular reflector traverse error, which can lead to the transverse shift of the spot.

Let *h* be the side length of the isosceles right triangle and the spot size set to 6 mm. The spot transverse displacement can be expressed as:

(17) $$\Delta Y_{2,3}\leq \frac{1}{2}(h-3)$$

- Triangular reflector angle error of the periscope array

**Figure 11:**
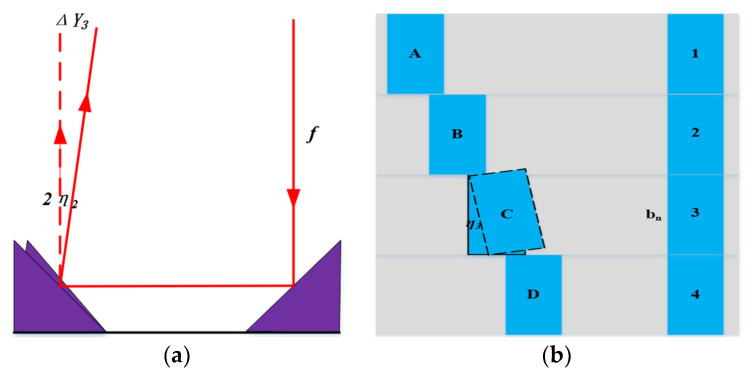
(**a**) Schematic diagram of pitch angle error of the triangular reflector. The arrow indicates the direction of light propagation. The dashed line represents the ideal propagation direction, and the solid line represents the actual propagation direction when there is an error; (**b**) schematic diagram of azimuth angle error of the triangular reflector, 1-4 represent different triangular reflectors, and A-D represent corresponding triangular reflectors, where C triangular reflector have an azimuth angle error.

As shown in Figure 11a, if the reflector of the periscope array has an angle error that is around the normal, then the spot offset can be expressed as:

(18) $$\Delta Y_{4}=2f\tan\eta_{2}$$

Figure 11b shows the schematic diagram of the top-view principal of a triangular reflector, and the spot offset can be expressed as:

(19) $$\Delta Y_{5}=b_{n}\tan\eta_{3}$$

The total error can be expressed as:

(20) $$\Delta Y=\sum_{i=1}^{5}Y_{i}$$

We use the distance between adjacent frame boundaries (1.5 mm) as the total error Δ*Y* constraint and, with comprehensive consideration of the sensitivity of the error and the level of fabrication, the error is distributed and tightened as shown in Table 3.

## 3. Results

**Figure 12:**
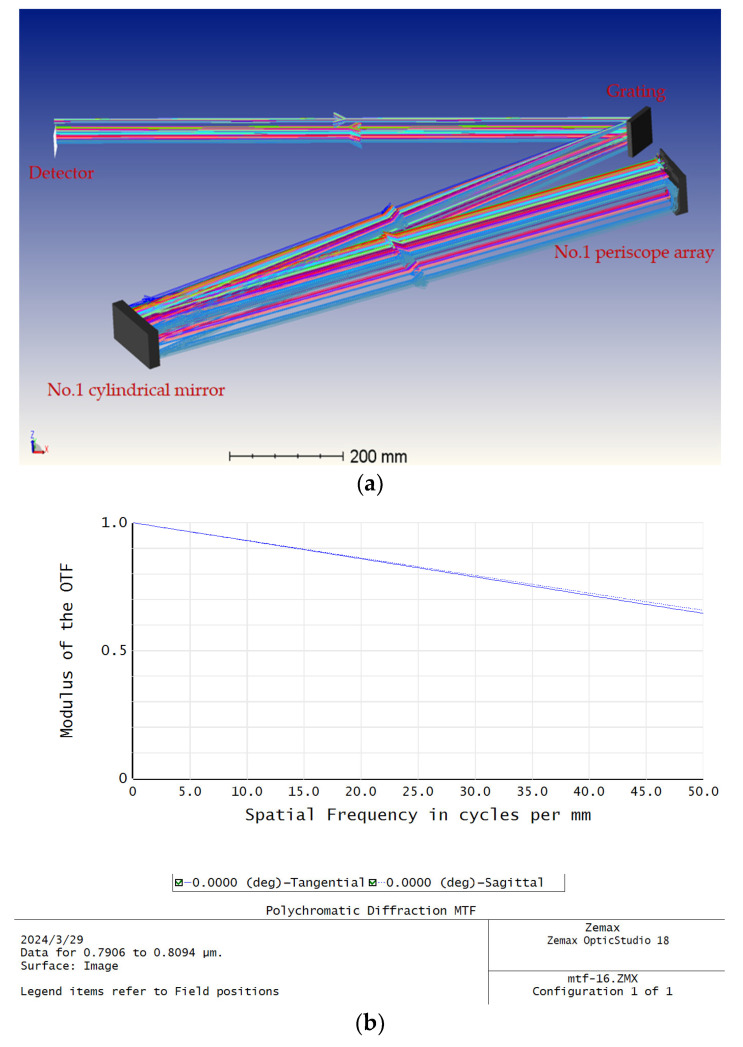

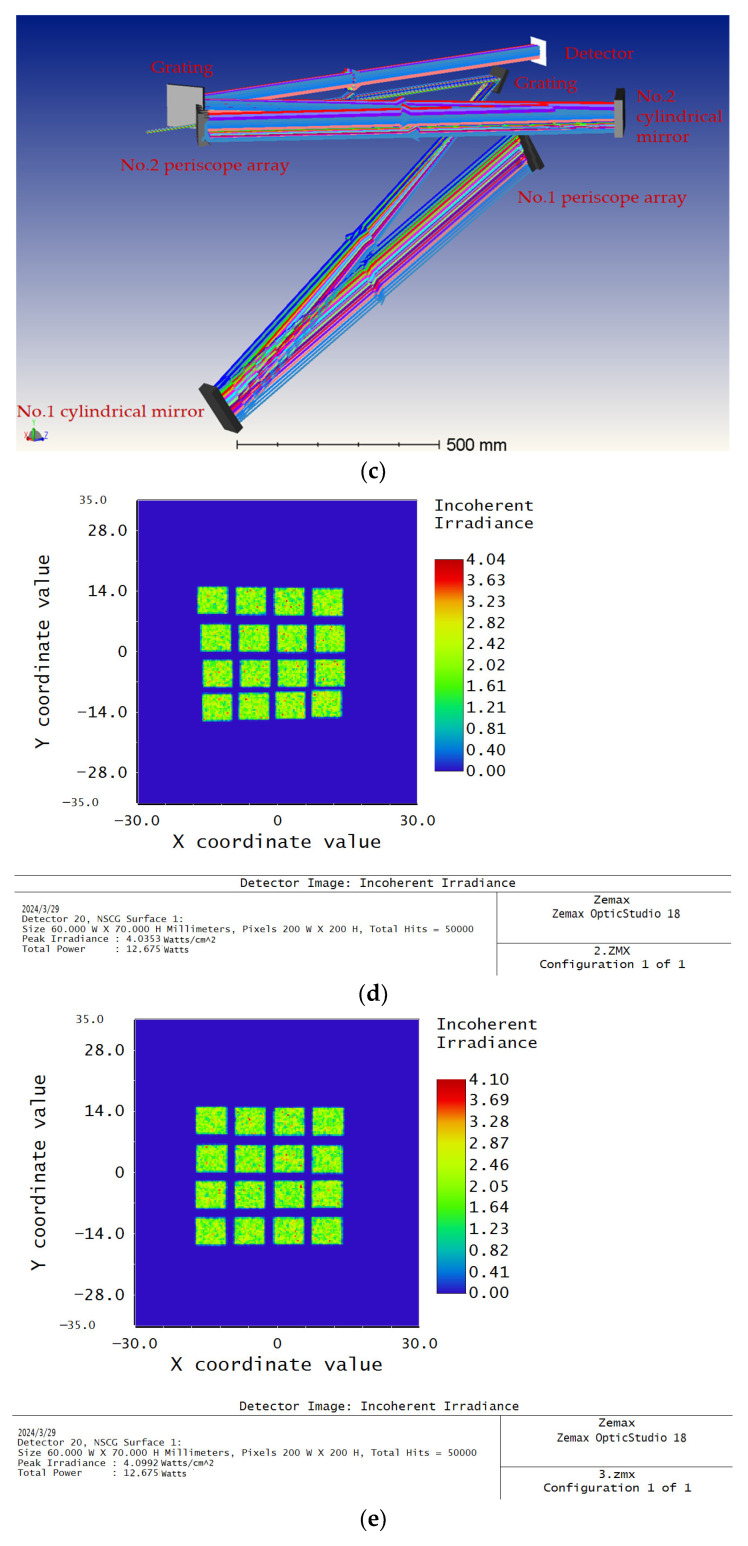
(**a**) Optical structure of one-dimensional (landscape orientation) all-optical spatial mapping module with different colors representing different wavelengths; (**b**) MTF curve of one-dimensional all-optical spatial mapping module; (**c**) optical structure of two-dimensional all-optical spatial mapping module with different colors representing different wavelengths; (**d**) spot diagram of two-dimensional all-optical spatial mapping module; (**e**) spot diagram of two-dimensional all-optical spatial mapping module after adjustment.

The technical parameters, such as in Table 1 and Table 2, are selected according to the above theoretical model, and the two-dimensional (2D) all-optical spatial mapping module is designed using ZEMAX software. A four-frame transverse module is constructed as shown in Figure 12a, and the MTF of the transverse module in sequence mode is shown in Figure 12b. It reaches 0.64 at 50 lp/mm, so the 50 lp/mm MTF of a two-dimensional module can be estimated to be above 0.3. The complete optical structure is shown in Figure 12c, and the spot distribution on the image plane of the complete optical structure is shown in Figure 12d. The design results show 1.25 nm spectral resolution and 16 wavelengths with the 4 × 4 frame arrangement on the detector. It should be explained that the unequal spacing of each frame is due to the non-linearity of grating dispersion. Fine tuning of the angle of the corresponding glass substrate of the periscope array allows for an approximate equidistant arrangement, as shown in Figure 12e.

The final result of our module design meets the indicators shown in Table 4:

## 4. Conclusions

To solve the problems of fewer frames, lower spectral resolution, and lower detector sensor utilization, a two-dimensional (2D) all-optical spatial mapping module is proposed, and the optical parameters model based on our optical structure is established. The theoretical analysis of the inter-frame energy crosstalk caused by incident beam spot width, chromatic aberration, and main errors of the periscope array has been conducted. The results show that an offset *w′* is equal to *w*/6, and crosstalk ratio is between 88% and 96%, which can better eliminate the effect of inter-crosstalk. The chromatic aberration caused by the width of the incident spot maximum value is 15.44 um, which is almost negligible. The angle error of the triangular mirror up to 0.01° is the most stringent among the fabrication errors of the periscope array. The design of our module can achieve a 16-frame image, 1.25 nm spectral resolution, and 0.3 at 50 lp/mm. Compared with the one-dimensional structure, the indexes of 6 frames and 3.33 nm spectral resolution are significantly improved, and almost equivalent to that of the slicing mirrors module’s 18 frames and 1.94 nm spectral resolution, while one ultra-fast image sensor was saved.

## Figures and Tables

**Table 1:** Main parameters of grating and cylindrical mirror.

Parameters	Value
Grating groove density	1800 g/mm
Incidence angle of grating	30°
Focal length of No. 1 cylindrical mirror	1067.5 mm
Focal length of No. 2 cylindrical mirror	1075 mm

**Table 2:** Value of b for the two periscope array.

Parameters	Value			
Value of b for the No. 1 periscope array	24.535 mm			
	26.133 mm			
	28.114 mm			
	30.653 mm			
Value of b for the No. 2 periscope array	6 mm	6.086 mm	6.177 mm	6.271 mm
	6.371 mm	6.475 mm	6.585 mm	6.702 mm
	6.825 mm	6.955 mm	7.093 mm	7.241 mm
	7.398 mm	7.566 mm	7.747 mm	7.942 mm

**Table 3:** Final numerical value of errors.

Parameters	Calculated Value	Final Value
Angle error of the glass substrate: *η*_1_	1.93°	0.5°
Traverse error of the triangular mirror: Δ*Y*_2_/Δ*Y*_3_	1.2 mm/1.2 mm	0.1 mm/0.1 mm
Angle error of the triangular mirror: *η*_2_/*η*_3_	0.0258°/1.06°	0.01°/0.1°

**Table 4:** Final indicator of two-dimensional all-optical spatial mapping module.

Indicator Name	Indicator Value
Number of frames	16 (4 × 4)
Size of single frame	6 mm × 6 mm
Spectral Bandwidth	20 nm (790–810 nm)
Spectral Resolution	1.25 nm
MTF@50 lp/mm	≥0.3

## Data Availability

Data are contained within the article and Supplementary Materials.

## Supplementary Materials

The following supporting information can be downloaded at: https://www.mdpi.com/article/10.3390/s24072219/s1. The optical structure of a two-dimensional (2D) all-optical spatial mapping module is stored as a Zemax file in the supplementary material.
